# Construct Validity of a Wearable Inertial Measurement Unit (IMU) in Measuring Postural Sway and the Effect of Visual Deprivation in Healthy Older Adults

**DOI:** 10.3390/bios14110529

**Published:** 2024-11-01

**Authors:** Luca Ferrari, Gianluca Bochicchio, Alberto Bottari, Alessandra Scarton, Francesco Lucertini, Silvia Pogliaghi

**Affiliations:** 1 Department of Neurosciences, Biomedicine and Movement Sciences, University of Verona, 37131 Verona, Italy; luca.ferrari_01@univr.it (L.F.); gianluca.bochicchio@univr.it (G.B.); alberto.bottari@univr.it (A.B.); alessandra.scarton@microgate.it (A.S.); 2 Department of Biomolecular Sciences, University of Urbino, 61029 Urbino, Italy; francesco.lucertini@uniurb.it; 3 Research Associate Canadian Center for Activity and Ageing, University of Western Ontario, London, ON N6A 3K7, Canada

**Keywords:** aging, postural control, balance, inertial sensors, risk of falls

## Abstract

Inertial Motor sensors (IMUs) are valid instruments for measuring postural sway but their ability to detect changes derived from visual deprivation in healthy older adults requires further investigations. We examined the validity and relationship of IMU sensor-derived postural sway measures compared to force plates for different eye conditions in healthy older adults (32 females, 33 males). We compared the relationship of the center of mass and center of pressure (CoM and CoP)-derived total length, root means square (RMS) distance, mean velocity, and 95% confidence interval ellipse area (95% CI ellipse area). In addition, we examined the relationship of the IMU sensor in discriminating between open- (EO) and closed-eye (EC) conditions compared to the force plate. A significant effect of the instruments and eye conditions was found for almost all the variables. Overall, EO and EC variables within (force plate r, from 0.38 to 0.78; IMU sensor r, from 0.36 to 0.69) as well as between (r from 0.50 to 0.88) instruments were moderately to strongly correlated. The EC:EO ratios of RMS distance and 95% CI ellipse area were not different between instruments, while there were significant differences between total length (*p* = 0.973) and mean velocity (*p* = 0.703). The ratios’ correlation coefficients between instruments ranged from moderate (r = 0.65) to strong (r = 0.87). The IMU sensor offers an affordable, valid alternative to a force plate for objective, postural sway assessment.

## 1. Introduction

Balance is a multidimensional concept referring to the ability of a person not to fall [1]. By the periodical monitoring of the age-related decline in balance, individuals with an increased risk of falls can be identified in a timely manner [2,3]. The early detection of the maintenance of balance is possible through the measure of postural control. The control of balance is associated with three different human activities: reaction to external disturbance (restoration), voluntary movement (achievement), and maintenance of a posture (maintenance) [1]. The latter is measurable through static sway, defined as the ability to maintain the center of pressure (CoP) within the limits of the base of support while standing still [4]. Static sway is one of the most studied balance components since it can be easily and safely estimated using clinical tests such as the Short Physical Performance battery test or the Berg balance scale [5,6]. Typically, these clinical tests are based on the visual assessment of the patient by using a scalar score [5,6]. Despite being flexible and easy to use, clinical tests are useful only for visible and gross balance deficits, excluding them as a tool for the early identification and monitoring of an increased risk of falls and/or the detection of subtle balance deficits [2].

To overcome these limitations of field tests, balance can be quantified objectively through posturography using optoelectronic systems or force plates [2,7]. Different useful variables can be extrapolated from the displacement of the CoP measured using the gold standard force plate method [4]. However, the well-known issues linked with the required equipment (high cost, needed for specialized personnel, not very transportable) limit the use of the gold standard equipment in laboratory settings only.

Therefore, alternative approaches have been sought to provide affordable yet objective measures that are also sensitive to change. In this context, instrumented clinical tests using inertial measurement units (IMUs) can provide large amount quantitative information about the individual’s sway. Typically, the IMU sensor is worn near the center of mass (CoM) at the lumbar spine level (on the L5 vertebra) and measures its tridimensional acceleration, velocity, and displacement [7,8]. Although these values could not be directly compared with the force plate-derived CoP variables [8,9], CoM and CoP are strictly related. Indeed, if the body moves like an inverted pendulum [10,11], a correlation close to 1 is expected between trunk acceleration and CoP displacement [9]. Therefore, a new wearable-based posturography method could be a valid alternative to the “classic” analysis of static sway [8]. Indeed, a recent systematic review [7] showed that IMU sensor-derived variables are moderate to highly correlated to CoP variables (r from 0.58 to 0.84 for mediolateral and anteroposterior sway) and are also able to distinguish between people of different ages (young vs. elderly) and health status (healthy individuals vs. Parkinson’s disease, multiple sclerosis, and other neurological conditions).

The ability to maintain balance control could be challenged by using different surfaces, reducing the base of support (i.e., feet position), or by sense deprivation (i.e., open and closed eyes), all conditions that can produce a greater magnitude of sway variables. In particular, visual deprivation is known to affect balance control negatively [3], and the ratio between closed- (EC) and open-eye (EO) conditions is frequently used to assess the visual contribution to the static sway ability. While it is well known that both force plate-derived CoP variables and IMU sensor-derived CoM variables are sensitive to the changes between EO and EC conditions [3,12,13] in a wide spread of neurological disorders [14], less is known about the ability of IMU sensors to detect these differences in healthy older individuals. To the best of our knowledge, no studies directly evaluated the relationship of the IMU sensor to detect changes in static sway variables due to visual deprivation compared to the gold standard instrument.

Therefore, the purpose of this study is twofold. In a group of healthy elderly individuals, we aimed to (i) evaluate the relationship between sway variables derived from an IMU sensor and derived from a force plate during static sway trials; (ii) evaluate the relationship between the variables derived from an IMU sensor and force plate in detecting CoM and CoP-related changes due to visual deprivation. We hypothesized that the IMU sensor and force plate-derived variables are strongly correlated and that the IMU sensor can detect changes in postural sway related to visual deprivation as the force plate.

## 2. Materials and Methods

### 2.1. Participants

A total of 65 healthy older individuals (32 females, 33 males) were recruited by local advertisement. The inclusion criteria were an age above 60 years, while a preliminary telephone interview and a successive medical screening allowed the exclusion of individuals with any orthopedic, mental, or neurological diseases that could interfere with the postural control. All participants signed a written informed consent form before participating. All procedures used in the study were approved by the Ethics Committee for Human Research from the University of Verona (28/2023) and were conducted in conformity with the Declaration of Helsinki.

### 2.2. Data Collection

Participants visited the laboratory one time. Personal (sex, age) and anthropometric (weight, height) data for each participant were first recorded. The anthropometric assessment was performed with participants barefoot and wearing only underwear. Body mass was taken to the nearest 0.1 kg with an electronic scale (Tanita electronic scale BWB-800 MA, Tokyo, JP), and stature was measured with a Harpenden stadiometer (Holtain Ltd., Crymych, Pembs., UK) to the nearest 0.5 cm. Body Mass Index (BMI) was calculated as body mass/height^2^ (kg/m^2^). Then, participants completed two (EO and EC conditions) 30 s standing balance tests with feet in a semi-tandem position (i.e., with the toe of the back foot in contact with the mid-front foot) [5]. The two tests were performed in randomized order.

For each test, all participants were instructed to stand upright with both feet on a single force plate (1000 Hz, AMTI Inc., Watertown, MA, USA), while, simultaneously, a single IMU sensor (500 Hz, GYKO, Microgate, Bolzano, Italy) was placed on the lumbar spine (i.e., L5 level) worn with the dedicated belt. According to the manufacturer’s user instructions, the IMU sensor was oriented into the belt’s pocket with the lead upward and outward, providing the *x*-axis to measure the anteroposterior displacement and the *y*-axis to measure the mediolateral displacement. The sensor’s height from the force platform’s surface was recorded and inserted in the dedicated software (GYKORePower Version 1.2.2.0, Microgate, Bolzano, Italy), which was in communication with the IMU sensor via Bluetooth^®^.

An operator showed the correct posture, which consisted of standing still with the feet in a semi-tandem position. There were no constraints on the arms’ position. After that, a familiarization session lasting 2 min was performed before each test. The same dedicated operator remained close to the participant to prevent any risk of falls while another operator monitored the instruments. The force plate and IMU sensor were manually synchronized after a countdown of three, and the trial recording automatically stopped after 30 s. The trial was interrupted and repeated if the participants moved their feet or grasped the operator for support.

### 2.3. Data Analysis

Regarding the COP-derived variables, raw data from the force plate were collected and subsequently analyzed using a self-written MATLAB code. Briefly, the force signal was low pass filtered at 5 Hz using a fourth-order Butterworth filter. After that, the total length, root mean square (RMS) distance, mean velocity, and 95% confidence interval ellipse area (95% CI ellipse area), as well as the anteroposterior (AP) and mediolateral (ML) component for each metric, were extracted following standard procedures [4].

The calculation of the IMU sensor is based on the inverse pendulum model [10,11], which relates the controlled variable CoM with the controlling variable CoP, stating that the difference between these two physical quantities is proportional to the CoM horizontal acceleration, and this relation holds in both the sagittal and frontal plane (i.e., the AP and ML direction) [11]. The model is based on two assumptions: (i) all the subject’s body mass is concentrated in one point (i.e., the CoM); (ii) the CoM is at the top of the inverse pendulum and is directly linked to the ankle joint by a rigid segment (the knee and hip joints are not considered). In a quiet state, the momentum applied on the CoM is counterbalanced by the active momentum applied by the ankle joint. Therefore, by using the accelerometer and gyroscope values and by knowing the height from the surface of the force plate (i.e., L5 to the surface of the force platform distance), it is possible to measure the horizontal displacement of the COM (in both directions).

The dedicated software automatically computed the CoM data from the IMU sensor, and the same CoP variables derived from the force plate were extracted. Finally, the ratios between variables derived from the EC and EO conditions were calculated [15].

### 2.4. Statistical Analysis

All data were calculated and reported as mean ± standard deviation. Shapiro–Wilk tests were run to test the normality of data distribution, and nonparametric tests were used when the assumption of normality was violated. A 2-way ANOVA was run to test differences between instruments and eye conditions. Pearson product–moment correlation (parametric data) or Spearman Rank correlation (nonparametric data) analyses were run to test the linear correspondences between eye conditions within the same instrument. Correlations were computed between instruments within the same eye condition to test the relationship between instruments in measuring the sway variables.

Finally, paired *t*-tests and correlations were also performed for the ratios of the most correlated variables between instruments. Correlation coefficients were interpreted as negligible (<0.1), weak (0.10 ≤ x < 0.40), moderate (0.40 ≤ x < 0.70), strong (0.70 ≤ x < 0.90), or very strong (≥0.9) [16]. Statistical significance was set at *p* < 0.05. SigmaPlot 12.5 (SigmaStat, San Jose, CA, USA) was used for all the statistical analyses.

## 3. Results

Participants’ anthropometric characteristics are reported in Table 1.

Absolute values of the variables, along with their within and between instrument correlation coefficients, are reported in Table 2 for both eye conditions.

ANOVA showed a statistically significant main effect of the instrument and eye conditions for all the variables except for the RMS distances, which did not display a main effect of the instrument on the ML axis for the OE condition and on both AP and ML axes for the CE condition. Overall, the variables for open- and closed-eye conditions derived from the instruments were weak–moderately to strongly correlated (force plate r, from 0.38 to 0.78; IMU sensor r, from 0.36 to 0.69). Moderate to strong correlations (r from 0.50 to 0.88) were also found between instrument variables for both eye conditions, except for the RMS distance in the mediolateral direction for the open-eye condition (r = 0.39).

**Figure 1:**
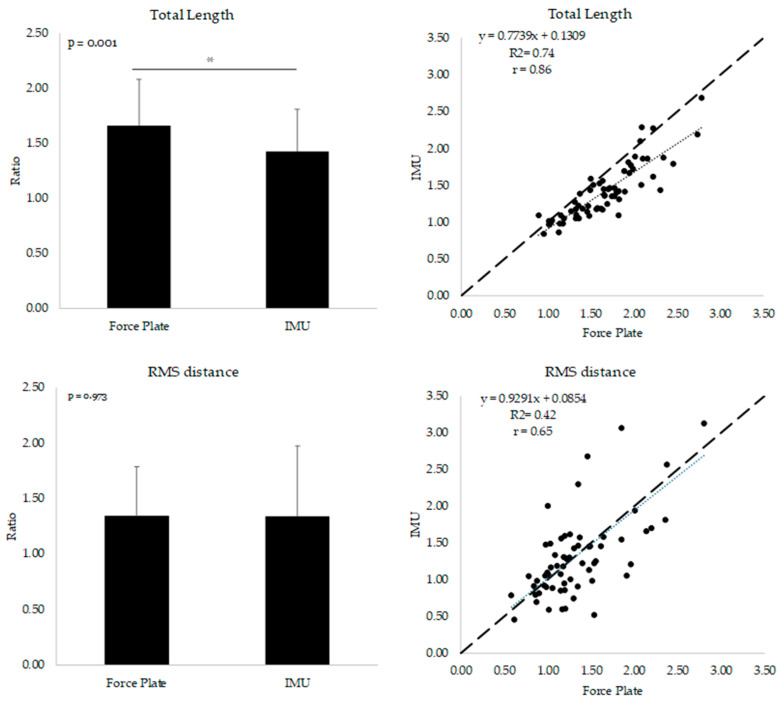

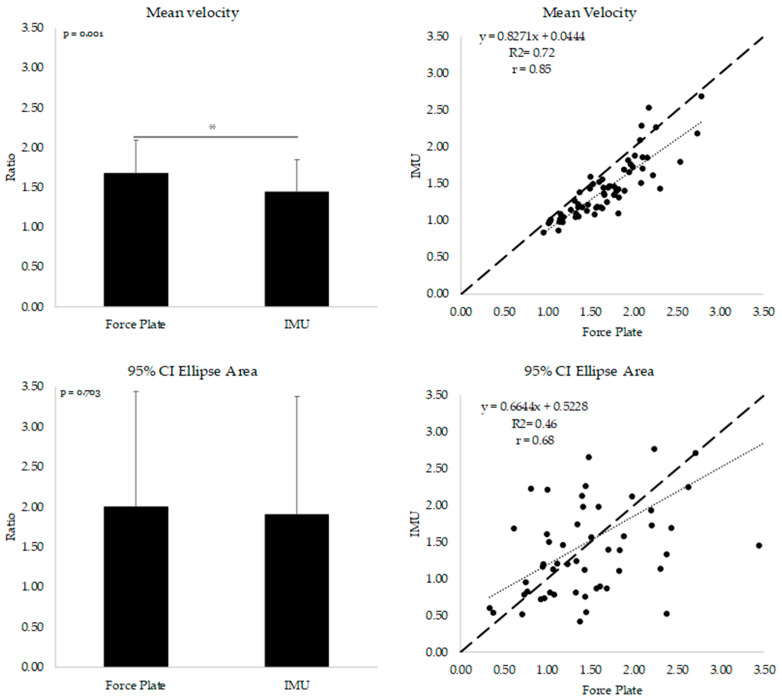
A comparison of the mean between ratios and correlation plots is displayed. * indicates statistical significance difference between means. Ratios were calculated by dividing the closed-eye values by the open-eye ones.

The comparison between means and correlation of the ratios derived from both instruments are displayed in Figure 1. The mean values of RMS distance and 95% CI ellipse were not different between instruments, while there was a significant difference between the total length and mean velocity variables (*p* = 0.973 and *p* = 0.703, respectively). Moderate to strong correlation coefficients were found for all the ratios (r from 0.65 to 0.87).

## 4. Discussion

The first aim of this study was to assess the relationship between static sway variables derived from an IMU sensor compared to a force plate in healthy older adults. Our results indicate that IMU sensor-derived variables showed close to moderate to strong agreement (r ranged from 0.38 to 0.87) with the gold standard instrument. The second aim of this study was to assess the ability of the IMU sensor to discriminate between open- and closed-eye trials compared to the force plate. Our findings confirmed that the variables derived from the open-eye trials using the IMU sensor were significantly different from those derived from the closed-eye trials (except for RMS distance in the anteroposterior direction). In addition, the IMU sensor showed moderate to strong agreement with the gold standard instrument for the EC and EO ratio across all the considered variables.

A direct comparison between the absolute values measured in our study with those reported in the literature is difficult because of a wide range of populations, foot positions, eye conditions, trial duration, and variables that have been analyzed in studies investigating static sway. When considering a similar foot position (semi-tandem), eye condition (open and closed), and the instrument used (force plate and IMU sensor), our values are comprehensively consistent with the literature regarding a population of healthy adults of both sexes [3,17,18,19,20,21]. The need to obtain valid, repeatable, and objective measures of static sway while using an affordable approach in clinical assessments has increased the attention toward wearable IMU sensors. Despite the appeal of these solutions, their actual validity and overall capability to track changes over time and between conditions have not yet been fully explored [8]. Some challenges arise from the assumptions underlying the use of IMU sensors for tracking the CoM. IMU sensors derive spatial, velocity, and area variables from CoM accelerations, whereas force plates measure variables based on the ground reaction force at the CoP. The inverted pendulum model linking the CoM and CoP through a rigid segment anchored at the ankle joint implies that every CoP displacement produces a related and proportional acceleration of the CoM, resulting in a correlation close to 1 [9,11]. However, while correct from a physics standpoint, this model overlooks the possible role of knee and hip joints, which can play a role in maintaining postural balance during quiet standing [9,11].

In addition, since IMU sensors and force plates measure different physical quantities, direct comparisons are impossible. Therefore, correlation analysis is commonly used to test the relationship between IMU sensors and force plate measures [22,23]. In agreement with previous work, our study found moderate to strong correlations for sway length (total length and in both directions: 0.50 to 0.88; RMS distance and in both directions: 0.39 to 0.75), velocity (0.48 to 0.88) and area (0.65 and 0.89) variables [7,24] (Table 2). The relationship between the IMU sensor and the force plate confirms that IMU sensors can be a valid solution for measuring static sway in healthy older adults. The availability of population-specific normative data on sway ability is essential for examining individual data, facilitating interpretation in support of decision-making and individualized exercise prescription (i.e., level- and goal-specific interventions). In this context, our study offers a medium-sized database of sway ability in healthy older individuals of both sexes.

The ability to maintain balance control is challenged by sense deprivation (i.e., open and closed eyes), which can lead to a greater magnitude of sway variables. In accordance with the literature [3], all the variables measured in our study were significantly greater in the EC compared to the EO condition for both force plates (absolute changes in total length +66%, RMS distance +30%, mean velocity +69%, and 95% CI ellipse area +72%) and IMU sensor-derived data (absolute changes in total length +43%, RMS distance +25%, mean velocity +45%, and 95% CI ellipse area +63%) (Tabel 2). The above absolute changes align with what is expected in this population of healthy older adults [3]. Moreover, in agreement with two previous studies that compared wearable IMU sensors to gold standard instruments, we confirmed a correlation between instruments within EC and EO conditions [24,25] (Table 2).

The relative change in the parameters obtained between EC and EO conditions is often used to assess proprioception-related neurologic disease by removing the visual and vestibular components contributing to balance maintenance [15]. In our study, we found ratios (expressed as % of EO condition) between variables derived from both instruments that are comparable to what was found in the literature for health- and age-matched individuals (from 134% to 201% and from 134% to 191% for the force plate and IMU sensor, respectively, vs. 147.4% ± 120.6%) [26,27] (Figure 1). In addition, our study was the first to directly focus on the ability of wearable IMU sensors to quantify the amplitude of the changes in sway parameters induced by the EC postural challenge, compared to gold standard instruments. The correlation coefficients (from 0.65 to 0.87, *p* < 0.001) showed that the ratios measured across all parameters were moderately to strongly related, highlighting the relationship between the variables derived from the instruments (Figure 1).

Tracking changes in postural sway over time is crucial when monitoring the healthy aging trajectory, and wearable sensors could be a valid, low-cost, and simple tool able to achieve this. However, this study did not investigate the IMU sensor’s ability to track changes over time. Future studies should investigate wearable sensors’ day-to-day reliability and sensitivity in detecting fine and subtle changes in postural sway. Moreover, practitioners and clinicians should be aware of the possible source of measurement error that arises from assessing postural sway under real-life conditions. Indeed, the moderate to strong correlations between IMU sensors and force plates in our study could be challenged outside the strictly controlled laboratory setting (i.e., clinical, home-based environments or self-administration). Among the sources of error that could arise from this type of setting, the strict control of the right posture during the sway assessment plays a key role. The biomechanical model (i.e., inverted pendulum model) on which the construct validity of the IMU sensor is based could be outflanked by postural sway strategies at the hip, knee, and ankle joints. For example, the pendulum’s length is measured by taking the height of the IMU sensor from the ground. If, during the sway trial, a subject slightly bends the knees, the final sway could result in less absolute values. Similarly, if a subject relies on hip strategies for maintaining static sway, the postulation that the inverted pendulum model is based on a rigid segment fails, and the resultant sway variables will be affected.

## 5. Conclusions

The current study examined the construct validity of an IMU sensor for postural sway assessment in healthy older individuals. Our results suggest that the IMU sensor is a valid alternative for postural sway assessment, as its CoM-derived measurements showed strong correlations with force plate-derived measurements. Moreover, this is the first study to investigate the ability of the IMU sensor to discriminate between trials with open and closed eyes in healthy older adults. The IMU sensor consistently distinguished and detected the expected differences in sway variables related to visual deprivation as the gold standard method. Therefore, the IMU sensor emerged as a valid option for evaluating sway and its perturbations induced by postural challenges in healthy older adults.

From a practical standpoint, the simple testing of the semi-tandem stance in both open- and closed-eye conditions, measured with an IMU device, could serve as a time-efficient yet valid tool for assessing sway ability in healthy individuals on a large scale. The relatively low cost and simplicity of use offer the opportunity to perform objective, digitalized, ample, and time-resolved measures in a clinical setting. This instrumented posturography could be used as a tool for the early detection and frequent monitoring of postural sway performance.

## Figures and Tables

**Table 1:** Anthropometric characteristics of the participants.

	Age (Years)	Weight (kg)	Height (m)	BMI (kg/m^2^)
Mean	66.7	73.1	1.68	25.9
Standard deviation	5.83	15.3	9.58	4.70
Minimum	60	48.9	1.52	17.5
Maximum	81	124	1.88	40.7

BMI: body mass index.

**Table 2:** Open and closed eye variables for both force plate and IMU sensor instruments, and correlation coefficients between instruments and between eye conditions within each instrument.

	EO Condition			EC Condition			EO-EC Correlation	
	Force Plate	IMU Sensor	r	Force Plate	IMU Sensor	r	Force Plate	IMU Sensor
Total length	796.8 ± 247.4	445.7 ± 104.5 †	0.62 ***	1322.5 ± 514.5 ^#^	637.5 ± 243.8 ^#^†	0.88 ***	0.73 ***	0.65 ***
AP	440.2 ± 156.1	241.1 ± 65.58 †	0.50 ***	736.6 ± 314.3 ^#^	355.9 ± 144.3 ^#^†	0.82 ***	0.68 ***	0.67 ***
ML	581.4 ± 177.4	324.9 ± 80.41 †	0.50 ***	961.3 ± 369.0 ^#^	456.1 ± 178.5 ^#^†	0.74 ***	0.72 ***	0.67 ***
RMS distance	9.2 ± 2.6	5.2 ± 1.7 †	0.58 ***	12.0 ± 3.8 ^#^	6.5 ± 2.7 ^#^†	0.75 ***	0.45 ***	0.36 *
AP	6.1 ± 2.3	6.0 ± 1.7 †	0.39 **	7.2 ± 2.6 ^#^	7.9 ± 3.1 ^#^	0.55 **	0.43 **	0.43 **
ML	6.8 ± 1.8	8.8 ± 3.4	0.43 ***	9.5 ± 3.1 ^#^	10.2 ± 4.4 ^#^	0.74 ***	0.38 **	0.43 **
Mean velocity	26.4 ± 8.25	14.8 ± 3.47 †	0.62 ***	44.7 ± 17.3 ^#^	21.5 ± 8.2 ^#^†	0.88 ***	0.78 ***	0.63 ***
AP	14.6 ± 5.20	8.05 ± 2.18 †	0.51 ***	24.9 ± 10.9 ^#^	12.0 ± 4.8 ^#^†	0.82 ***	0.74 ***	0.64 ***
ML	19.3 ± 5.92	10.8 ± 2.68 †	0.48 ***	32.3 ± 12.1 ^#^	15.4 ± 6.0 ^#^†	0.75 ***	0.75 ***	0.69 ***
95% CI ellipse area	876.0 ± 594.0	88.5 ± 48.9 †	0.65 ***	1507.6 ± 963.2 ^#^	144.5 ± 101.8 ^#^†	0.89 ***	0.45 **	0.44 ***

EO, open eye; EC, closed eye; IMU, inertial measurement unit; AP, anterior–posterior; ML, mediolateral; RMS, root mean squared. # indicates significant difference between eye conditions; † indicates significant difference between instruments; * indicates significant relation: * *p* < 0.05; ** *p* < 0.01; *** *p* < 0.001.

## Data Availability

The data presented in this study are available upon request from the corresponding author. The data are not publicly available due to restrictions (privacy).
